# Comparison of periarticular injection and low-concentration high-volume suprainguinal fascia Iliaca plane block in total knee arthroplasty: a randomized prospective study

**DOI:** 10.1007/s00402-025-05962-1

**Published:** 2025-06-21

**Authors:** Mehmet Cenk Turgut, Elif Oral Ahiskalioglu, Yunus Emre Karapinar, Ela Medetoglu Koksal, Cagatay Engin, Erkan Cem Celik, Muhammed Enes Aydin, Ahmet Murat Yayik

**Affiliations:** Atatürk University, Erzurum, Turkey

**Keywords:** Suprainguinal fascia Iliaca plane block, Periarticular infiltration, Total knee arthroplasty

## Abstract

**Background:**

The suprainguinal fascia iliaca plane block (SFIPB) has been used effectively for postoperative analgesia in hip surgeries due to its extensive dermatomal coverage. This technique may also serve as an alternative in knee surgeries. However, studies investigating the use of SFIPB in total knee arthroplasty (TKA) are limited. This study evaluates the efficacy of SFIPB compared to periarticular infiltration (PAI) in TKA, focusing on postoperative opioid consumption, pain scores, motor function, and rehabilitation outcomes.

**Methods:**

This randomized controlled trial included 70 patients undergoing TKA, allocated to either the SFIPB group or the PAI group. Postoperative pain management was standardized across groups using patient-controlled fentanyl analgesia. Primary outcomes included opioid consumption over 48 h, and secondary outcomes assessed pain scores, quadriceps strength, mobilization times, range of motion (ROM), and rehabilitation test results.

**Results:**

SFIPB resulted in significantly reduced opioid consumption at 24 and 48 h compared to PAI (*p* < 0.001). Pain scores assessed via visual analogue scale (VAS) were lower in the SFIPB group, particularly for anterior knee pain during rest and movement (*p* < 0.05). SFIPB also demonstrated superior rehabilitation outcomes, with improved quadriceps strength (*p* = 0.002) and better performance on the Time Up and Go (TUG) test. Both groups reported minimal side effects, but opioid-related nausea and vomiting were less frequent in the SFIPB group.

**Conclusion:**

The use of SFIPB with a dilute, high-volume local anesthetic was superior to PAI by reducing opioid consumption and opioid-related side effects without impairing motor function or patient mobilization. This highlights its potential as an effective analgesic technique in TKA.

## Introduction

Total knee arthroplasty (TKA) is a common and effective surgical procedure aimed at restoring joint function and alleviating pain in patients with severe knee joint damage. This procedure involves the replacement of the medial and lateral femorotibial joints as well as the patellofemoral joint and is often associated with significant postoperative pain. Effective postoperative pain management is crucial for the success of TKA, as it can enhance patient recovery, facilitate early mobilization, and reduce the length of hospital stay. Current postoperative analgesia strategies include a multimodal approach, utilizing opioids, nonsteroidal anti-inflammatory drugs, gabapentinoids, peripheral nerve blocks, and periarticular injections to achieve optimal pain control [1].

The suprainguinal fascia iliaca block (SFIPB), which has recently gained traction in hip surgeries, is an advanced regional anesthesia technique providing effective analgesia by targeting the femoral, lateral femoral cutaneous, and obturator nerves [2]. While its primary application has been in hip arthroplasty, the unique anatomical and functional characteristics of SFIPB render it a promising option for TKA within a multimodal analgesia framework [3]. This is attributed to the block’s ability to inhibit afferent sensory transmission from the overlapping dermatomal distributions of the femoral, lateral femoral cutaneous, and obturator nerves, which extend beyond the hip joint to contribute to knee innervation. Consequently, the inclusion of SFIPB in the perioperative analgesic regimen for TKA has the potential to enhance postoperative pain management, minimize opioid consumption, and improve overall functional outcomes [4].

In parallel, periarticular infiltration (PAI) analgesia remains a cornerstone in TKA pain management strategies. This technique, characterized by the direct deposition of local anesthetics into the periarticular tissues surrounding the knee joint, has consistently demonstrated efficacy in providing robust postoperative analgesia and facilitating early mobilization in patients undergoing TKA [5].

The primary objective of this study is to compare the effects of SFIPB and PAI on postoperative opioid consumption in patients undergoing TKA. Secondary objectives include evaluating the impact of these analgesic techniques on postoperative pain scores, TUG test results, range of motion (ROM) assessments, five times sit-to-stand test (FTSST) outcomes, and mobilization times. By elucidating the comparative efficacy of these analgesic modalities, this study aims to contribute to the optimization of postoperative pain management protocols in TKA, ultimately enhancing patient outcomes and recovery. This randomized, prospective clinical trial is designed to evaluate evidence-based analgesic strategies and improve postoperative recovery in TKA.

## Materials and Methods

**Fig. 1:**
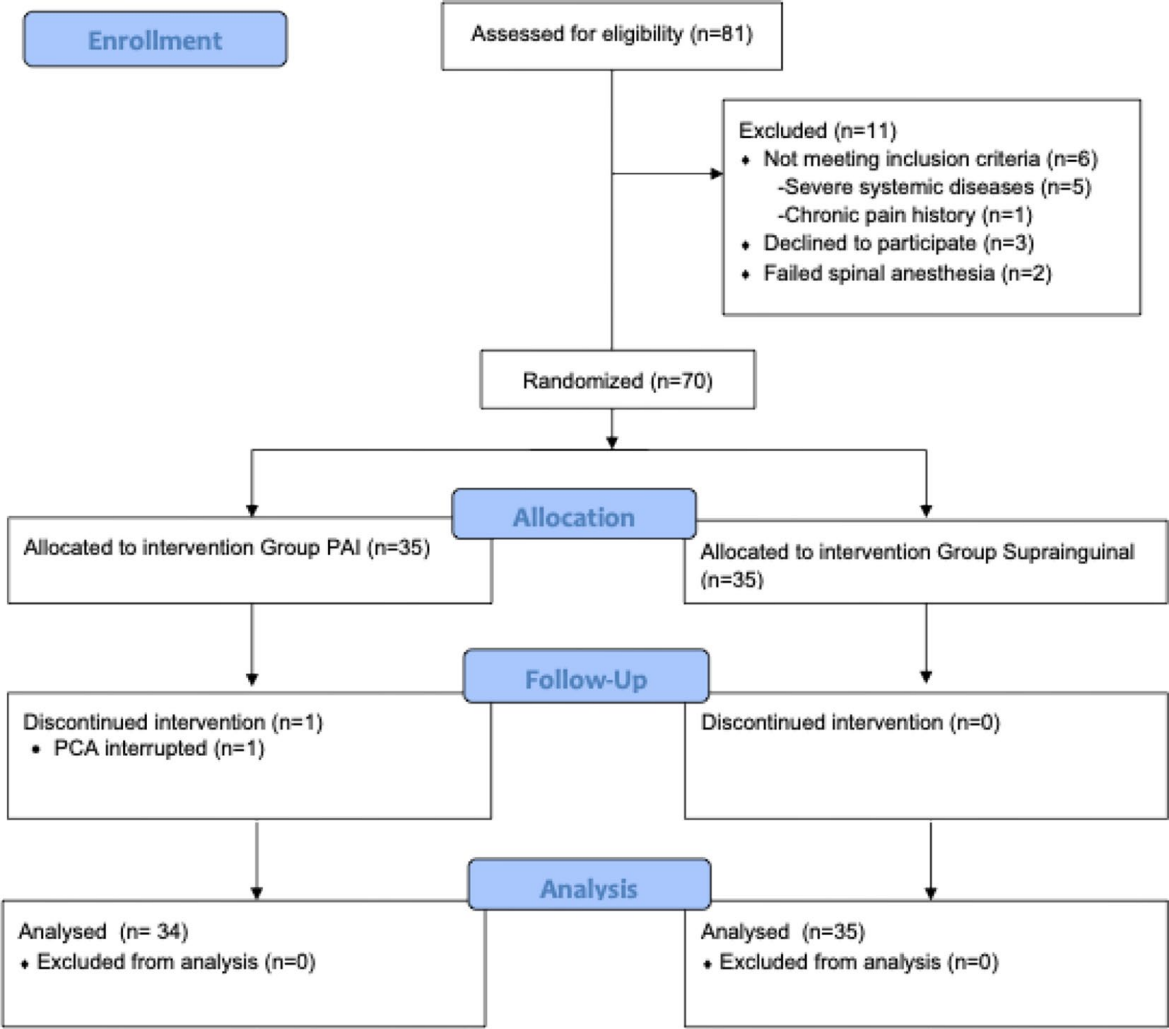
CONSORT diagram of the study

This randomized controlled trial was carried out at *** University from November 25, 2022, to May 15, 2023, adhering to the Declaration of Helsinki principles. Approval was granted by the local ethics committee (***). Before the study commenced, it was prospectively registered on clinicaltrials.gov (***). The CONSORT diagram illustrating the study design is shown in Fig. 1, aligning with relevant guidelines. Written informed consent was obtained from all participants for both their involvement in the study and the interventions applied. Following approval from the local ethics committee, 70 patients aged 18–65 years with ASA physical status I-III, without significant hepatic, renal, or advanced cardiac failure, and not on regular analgesic medication or having used analgesics in the last 24 h, were enrolled. Patients with severe cardiovascular disease, liver dysfunction, coagulopathy, anticoagulant usage, non-cooperative behavior, allergies to study drugs, or those unwilling to participate were excluded.

### Randomization and blinding

Randomization was performed using Microsoft Excel (Office 365, Microsoft Corp., WA, USA). Eligible patients provided written informed consent prior to study enrollment. One hour prior to surgery, participants were randomly assigned to either the SFIPB or PAI group using a randomization table generated with the “RAND” function. The patient study numbers and group allocations were concealed in sealed opaque envelopes and delivered to the operating theatre technician, who prepared the respective interventions.

Blinding procedures were implemented as follows: the outcome assessor, responsible for recording postoperative pain scores, rehabilitation test results, and rescue analgesic use, was blinded to group allocation. Patients were also blinded to the intervention they received. Due to the nature of the procedures, the anesthesiologist administering the regional block and the surgeon performing the periarticular injection could not be blinded. However, these individuals did not participate in outcome assessments. This approach ensured that the data collection and analysis were performed without knowledge of group assignment, thereby minimizing assessment bias.

### Application of spinal anesthesia and surgical procedure

Upon entering the operating room, standard monitoring, including electrocardiography, pulse oximetry, and non-invasive arterial blood pressure measurement, was applied to all patients. Spinal anesthesia was administered using a sterile technique. Patients were placed in a sitting or lateral decubitus position. A 25-gauge spinal needle was then inserted into the subarachnoid space, confirmed by the free flow of cerebrospinal fluid at the L3-L4 or L4-L5 interspace. Hyperbaric bupivacaine (15 mg) was administered intrathecally to achieve the desired level of anesthesia. Following the administration of spinal anesthesia, patients were positioned supine. Before the administration of spinal anesthesia, patients received 8 mg of ondansetron and 8 mg of dexamethasone intravenously.

### Application of SIFIB block

**Fig. 2:**
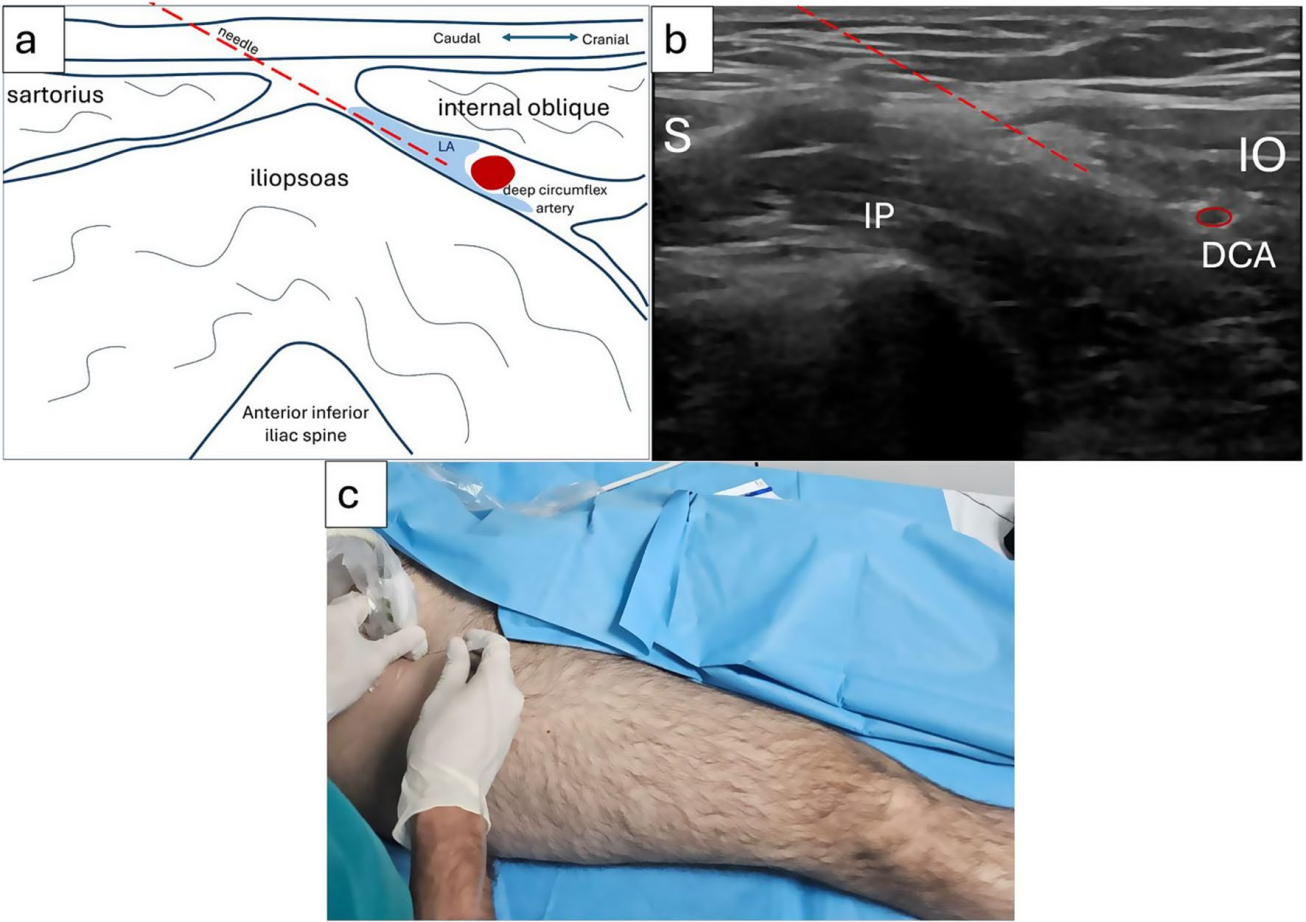
a) Sono anatomic illustration of suprainguinal fascia iliaca plane block with needle and local anesthetic distribution. (LA: local anesthetic) b) Ultrasound image illustrating the suprainguinal fascia iliaca plane block puncture trajectory. c) Patient-probe and needle orientation for block application

Patients were randomly assigned to two groups: Group 1 received SFIPB and Group 2 received PAI analgesia. Group 1 underwent SFIPB following spinal anesthesia. The linear ultrasound probe was placed medial to the anterior superior iliac spine, moving inferomedially along the inguinal ligament until the deep circumflex artery was visualized. A 100 mm needle was advanced from caudal to cranial, reaching the fascia iliaca under hydro-dissection to separate it from the iliacus muscle, creating a space for the injection of a local anesthetic mixture (20 ml 0.5% bupivacaine, 1:200,000 epinephrine, and 40 ml isotonic saline, totaling 60 ml).(Fig. 2).

### Application of PAI

Group 2 received PAI performed by orthopedic surgeons, with a mixture of 150 mg bupivacaine, 0.3 mg epinephrine, 30 mg ketorolac, and 50 ml normal saline, totaling 80 ml. This mixture was injected into the medial and lateral capsule, medial and lateral meniscus borders, deep part of the medial ligament, medial and lateral synovial recesses, and patellar ligament and quadriceps tendon.

### Surgical procedure

After identifying the landmarks, a midline longitudinal incision is made, followed by subcutaneous dissection and medial parapatellar arthrotomy. The patella is dislocated and everted, the knee flexed, and the anterior cruciate ligament, along with the anterior horns of both menisci are excised. Osteophytes are removed, and the femoral canal is accessed via intramedullary drilling. The distal femur is cut using an intramedullary guide, followed by anterior and posterior resections guided by an oscillating saw. Tibial cuts were performed using an extramedullary alignment guide in all patients. Patellar resurfacing was not performed routinely in all cases but was selectively applied based on intraoperative assessment. Specifically, patellar resurfacing was performed in patients presenting with significant retropatellar cartilage degeneration, including those with a history of rheumatoid arthritis, a dysplastic or flattened patella, or in patients younger than 60 years of age. After assessing the extension gap, trial components are inserted to evaluate knee flexion and extension, then removed. Cement is applied to the tibial and femoral implants before final component placement. A pneumatic tourniquet was utilized in all procedures to maintain a bloodless surgical field. Elastic tourniquets were not employed. The tourniquet was deflated following cement polymerization and prior to wound closure to allow for adequate hemostatic control. Intravenous tranexamic acid was administered at a dose of 10 mg/kg (maximum 1 g) approximately 10 min before skin incision, and the same dose was repeated during wound closure to minimize perioperative blood loss. The arthrotomy is sutured, the skin is closed in layers, and a sterile dressing is applied.

### Standard perioperative analgesia protocol and Follow-Up of pain

Preoperative assessments included walking aid usage, varus-valgus deformity, VAS scores (anterior and posterior), TUG test, quadriceps muscle strength at 0°, 45°, and 90° knee flexion, and active ROM. The TUG test was used to evaluate basic functional mobility by measuring the time taken to rise from a chair, walk 3 m, return, and sit down. Quadriceps strength was assessed isometrically at specified flexion angles to capture force variability across the functional arc. Active ROM was measured using a goniometer and reflected the degree of voluntary joint movement, serving as a marker of preoperative mobility and pain limitation. These results were recorded.

Standard monitoring (electrocardiography, pulse oximetry, and noninvasive arterial blood pressure) was applied once patients were in the operating room. Prior to spinal anesthesia, patients received 8 mg ondansetron and 8 mg dexamethasone intravenously. Postoperative analgesia was managed with intravenous patient-controlled analgesia using fentanyl at a concentration of 10 mcg/ml, with a loading dose of 50 mcg, a 15-minute lockout interval, and a 25-mcg bolus without basal infusion, continued for 24 h. In the recovery room, patients with a VAS score of 4 or higher received 25 mg meperidine. Additionally, all patients received scheduled non-opioid analgesics consisting of paracetamol 1 g four times daily and deksketoprofen 50 mg twice daily to optimize multimodal analgesia. Pharmacologic prophylaxis for deep vein thrombosis was administered using subcutaneous enoxaparin 40 mg once daily, initiated on the first postoperative day and continued for 15 days in all patients, following a standardized institutional protocol.

### Outcome measurements

Postoperative pain assessments were conducted at 1, 2, 4, 8, 12, and 24 h. Data collected included postoperative opioid consumption, time to first analgesia, additional analgesic consumption, nausea, vomiting, sedation, itching, drug-related side effects, and block-related complications. Mobilization time, VAS scores, TUG test results, quadriceps muscle strength, ROM, and FTSST outcomes were evaluated and recorded on the first and second postoperative days. The FTSST, a functional mobility assessment, measures the time required for a patient to rise from a chair and sit back down five times as quickly as possible without using their arms, reflecting lower extremity strength, balance, and functional independence.

### Sample size and statistical analyze

Sample size estimation was based on preliminary data assessing 48-hour opioid consumption in two groups of seven patients each (Group SFIPB: 353.57 ± 221.93 vs. Group PAI: 539.28 ± 212.55). To achieve a Type I error rate of 0.05, an effect size of 0.859, and a power of 0.85, a minimum of 26 participants per group was calculated. Anticipating a 20% potential dropout rate, the target sample size was increased to 35 patients per group to ensure adequate power.

Statistical analyses were conducted using SPSS software (version 20). The normality of data distribution was assessed with the Kolmogorov-Smirnov test and visual inspection of histograms. Continuous variables not following a normal distribution were analyzed using the Mann-Whitney U test, whereas normally distributed data were compared using the independent samples t-test. Categorical variables were evaluated using Fisher’s exact test or the chi-square test as appropriate. A p-value < 0.05 was considered indicative of statistical significance.

## Results

Among the 81 eligible patients, 11 were excluded for not meeting the inclusion criteria, leaving 70 patients who were randomized into two groups. Subsequently, one patient in the PAI group was excluded due to an interruption in patient-controlled analgesia, resulting in 34 patients being analyzed in the PAI group and 35 patients in the Suprainguinal group. The participant flow is illustrated in Fig. 1. As detailed in Table 1, no statistically significant differences were observed between the groups regarding demographic characteristics and intraoperative variables (*p* > 0.05).

**Table 1:** Demographic and Intraoperative Characteristic of Study Patients

		Group PAI *n* = 34	Group Suprainguinal *n* = 35	p
Age (y)		63.50±3.26	63.06±3.57	0.868^a^
Gender (m/f)		7/27	8/25	0.827^b^
BMI (kg/m^2^)		31.53±3.34	31.10±3.07	0.578^a^
ASA (II/III)		31/3	35/0	0.114^c^
Operation time (min)		86.62±21.13	94.14±20.45	0.137^a^
Anesthesia time (min)		102.79±20.75	11.43±20.20	0.084^a^
Walking aid (yes/no)		6/26	3/32	0.306^c^
Deformity	No	26	24	0.346^d^
	Varus	8	9	
	Valgus	0	2	

Values are presented as a number or mean ± standard deviation. Abbreviations, PAI: periarticular injection BMI: body mass index; ASA: American Society of Anesthesiologists physical status classification system aYate’s continuity correction, bIndependent Samples Test, cFisher’s exact test, dChi-square test

Patients in the SFIPB group demonstrated reduced opioid consumption compared to the PAI group across multiple time intervals (0–4 h, 4–8 h, and total 24 and 48 h; *p* < 0.05) (Table 2). Cumulative opioid consumption over 48 h was significantly lower in the SFIPB group (380.00 ± 177.59 mcg) compared to the PAI group (572.06 ± 233.61 mcg; *p* < 0.001). Time to first rescue analgesic requirement was prolonged in the SFIPB group (304.29 ± 232.43 min) versus the PAI group (185.29 ± 54.22 min; *p* = 0.005).

Postoperative pain scores, assessed using VAS, were significantly lower in the SFIPB group at both rest and movement for anterior knee pain (*p* < 0.05). Additionally, quadriceps muscle strength was superior in the SFIPB group (*p* = 0.002), and rehabilitation outcomes, including the TUG test on postoperative days 1 and 2, showed significantly better performance in the SFIPB group (*p* < 0.01). Comparison of postoperative VAS scores and rehabilitation measures are presented in Table 3. Quadriceps muscle strength, ROM, and functional outcomes measured by the TUG and FTSST demonstrated improved recovery trajectories in the SFIPB group, with significant differences in TUG performance on postoperative days 1 (*p* = 0.009) and 2 (*p* = 0.006) and ROM on day 2 (*p* = 0.004). No statistically significant differences in VAS scores were observed between the SFIPB and PAI groups during both rest and movement across nearly all time points in the postoperative period (Table 4). The comparison of preoperative VAS scores and rehabilitation measures revealed no statistically significant differences, as shown in Table 5. Side effects were minimal in both groups; however, opioid-related nausea and vomiting were less frequent in the SFIPB group (Table 6).

**Table 2:** Opioid Consumptions, Rescue Analgesia and First Rescue Requirement

	Group PAI *n* = 34	Group Suprainguinal *n* = 35	p^a^
0–4 h (mcg)	69.85±48.76	26.43±29.04	< 0.001*
4–8 h (mcg)	97.79±84.02	55.71±42.91	0.012*
8–24 h (mcg)	162.50±127.96	112.14±82.33	0.058
Total 24 h (mcg)	327.20±183.54	202.86±131.84	0.002*
Total 48 h (mcg)	572.06±233.61	380.00±177.59	< 0.001*
Rescue analgesia (yes/no)	19/15	21/14	0.729^b^
First rescue analgesic requirement time (min)	185.29±54.22	304.29±232.43	0.005*
Total rescue tramadol dose in 48 h (mg)	114.71±115.82	91.43±85.31	0.344

Values are presented as a number or mean ± standard deviation. Abbreviations, PAI: periarticular injection aIndependent Samples Test, bChi-square test

**Table 3:** Comparison of Preoperative Visual Analogue Scale Scores and Rehabilitation Measures

	Group PAI *n* = 34	Group Suprainguinal *n* = 35	p^a^
At movement			
Anterior	6.50 ± 1.96	6.23 ± 2.09	0.579
Posterior	4.94 ± 2.00	5.40 ± 2.34	0.384
At rest			
Anterior	4.38 ± 1.81	4.49 ± 1.77	0.811
Posterior	3.00 ± 1.78	3.94 ± 2.13	0.50
Quadriceps strength	4.71 ± 0.68	4.89 ± 0.32	0.167
TUG (sec)	17.18 ± 4.60	17.74 ± 4.18	0.594
ROM (°)	106.32 ± 19.04	110.86 ± 13.37	0.258

Values are presented as a number or mean ± standard deviation. Abbreviations, PAI: periarticular injection; TUG: time up and go test; ROM: range of motion aIndependent samples test

**Table 4:** Comparison of Postoperative Visual Analogue Scale Scores and Rehabilitation Measures

	Group PAI *n* = 34	Group Suprainguinal *n* = 35	p^a^
At movement			
Anterior	3.44 ± 2.31	1.97 ± 1.47	0.003*
Posterior	3.08 ± 2.17	3.20 ± 1.51	0.805
At rest			
Anterior	2.24 ± 1.92	1.14 ± 0.94	0.005*
Posterior	2.24 ± 2.05	2.23 ± 1.19	0.987
Quadriceps strength	3.50 ± 0.93	4.14 ± 0.69	0.002*
Mobilization time (h)	12.35 ± 6.39	14.39 ± 5.55	0.143
FTSST (sec)	52.44 ± 14.79	49.12 ± 8.54	0.244
TUG (sec)			
1st day	48.79 ± 15.59	39.89 ± 11.23	0.009*
2nd day	40.94 ± 14.37	32.66 ± 9.16	0.006*
ROM (°)			
1st day	74.76 ± 18.68	81.71 ± 12.00	0.072
2nd day	87.35 ± 16.01	96.86 ± 9.32	0004*

Values are presented as a number or mean ± standard deviation. Abbreviations, PAI: periarticular injection; FTSST: five times sit to stand test; TUG: time up and go test; ROM: range of motion aIndependent samples test

**Table 5:** Comparison of Postoperative Visual Analogue Scale Scores

	Group PAI *n* = 34	Group Suprainguinal *n* = 35	p^a^
At movement			
PACU	0 (0–0)	0 (0–0)	0.310
1 h	0 (0–0)	0 (0–0)	0.081
2 h	0 (0–0)	0 (0–0)	0.003*
4 h	2 (1–3)	2 (0–3)	0076
8 h	3 (2–3)	3 (2–3)	0.990
12 h	3 (2–4)	3 (2–3)	0.238
24 h	3.5 (2–6)	3 (3–4)	0.514
At rest			
PACU	0 (0–0)	0 (0–0)	0.310
1 h	0 (0–0)	0 (0–0)	0.142
2 h	0 (0–0)	0 (0–0)	0.022*
4 h	1 (1–2)	1 (0–2)	0.067
8 h	2 (1–2)	2 (1–2)	0.931
12 h	2 (1–3)	2 (1–2)	0.571
24 h	2 (1–4)	2 (2–3)	0.602

NOTE. Values are presented as median (IQR). Abbreviations, PAI: periarticular injection; PACU: post anesthesia care unit aMann-Whitney U test

**Table 6:** Incidence of Adverse Events

	Group PAI *n* = 34	Group Suprainguinal *n* = 35	p^a^
Nausea and vomiting	9	2	0.023*
Constipation	2	0	0.239
Itching	2	0	0.239
Urinary Retention	1	0	0.493
Xerostomia	0	0	N/A

Values are presented as numbers. Abbreviations, PAI: periarticular injection aFisher’s Exact Test

## Discussion

Our study demonstrated that when compared to PAI, low-dose high volume SIFIB provided superior analgesia by reducing opioid consumption and opioid related side effects without affecting motor outcomes and patient mobilization.

TKA remains a cornerstone in orthopedic surgery, providing significant improvements in joint function and quality of life [6]. However, the severe postoperative pain associated with TKA necessitates effective analgesic strategies to alter their ability to perform physical exercise and delay their recovery process.

The combination of multimodal analgesic regimens with regional anesthetic techniques provides better pain control following TKA, reduces opioid-related side effects, and shortens hospital length of stay [7]. While implementing these combinations, the primary objective should be to achieve optimal analgesia while minimizing motor blockade. Among motor-sparing blocks utilized in knee surgery, the adductor canal and femoral triangle blocks are commonly employed for the anterior aspect of the knee, while the infiltration between the popliteal artery and capsule of the knee (IPACK) and popliteal plexus blocks target the posterior region. Furthermore, genicular nerve blocks and periarticular infiltration techniques performed by surgeons significantly contribute to the overall analgesic efficacy, offering a comprehensive approach to pain management.

Recent clinical practice guidelines issued by leading surgical and orthopedic societies, as well as anesthesiology societies such as American Society of Regional Anesthesia and Pain Medicine, European Society of Regional Anaesthesia and Pain Therapy, and the Procedure Specific Postoperative Pain Management working group, strongly advocate for the use of intraoperative PAI to alleviate postoperative pain and reduce opioid consumption following primary total knee arthroplasty. The administration of a peri-articular cocktail injection is an efficacious method for attaining enhanced pain management during the early post-operative phase. Although the benefits are not enduring, their superior effects can be leveraged for early functional recovery following TKA and enhanced patient rehabilitation [1, 8, 9]. Intraoperative PAI provides limited analgesic efficacy and demonstrates its peak effectiveness within the first 12 h following TKA [10, 11]. The synergistic effect of a single-injection nerve block combined with PAI enhances analgesic efficacy, reduces opioid requirements, and accelerates postoperative physical performance compared to either intervention alone. In our study, the efficacy of PAI as a standalone SFIPB. Although PAI demonstrated a beneficial effect on pain scores, its impact on reducing opioid consumption and the requirement for additional analgesics was notably limited when used independently. These findings underscore the significant advantages of combination approaches in achieving effective postoperative analgesia. (Table 2)

In the context of knee surgery, achieving motor-sparing analgesia remains a critical goal in optimizing postoperative recovery [12]. To prevent quadriceps weakness, several novel peripheral nerve blocks have been introduced, and modifications of existing techniques have been implemented. These modifications include reducing the volume of local anesthetic, lowering the concentration of the local anesthetic, or choosing more distal nerve blocks. In our study, we aimed to minimize motor blockade by utilizing a high volume (60 mL) of low-concentration local anesthetic. When early recovery was assessed using the TUG and TSST, no significant differences were observed compared to the PAI group. This finding highlights the motor-sparing effect of this protocol. Moreover, these findings suggest that high volume-low concentration SFIPB supports early mobilization, a critical factor in reducing the risk of thromboembolism and facilitating rehabilitation.

The SFIPB has traditionally been employed for postoperative analgesia in hip surgery. However, its application in knee surgery remains relatively underexplored. Consistent with recent literature, our findings indicate that SFIPB effectively reduces postoperative opioid consumption. This aligns with the results of Kefeli Çelik et al., who demonstrated decreased morphine requirements in patients receiving SFIPB compared to control groups following TKA [4]. Moreover, Genc et al.’s dose-finding study highlighted the utility of low-concentration bupivacaine in minimizing motor weakness while achieving effective analgesia [13]. These findings align with our data, where SFIPB significantly decreased opioid-related side effects, including nausea, compared to PAI, supporting its utility in multimodal analgesia protocols. In our study, PONV was significantly less frequently seen in the SFIPB group. This difference may be attributed to the reduced opioid consumption observed in the SFIPB group, as opioid-related side effects such as nausea and vomiting are dose-dependent. The superior analgesic efficacy of SFIPB likely contributed to diminished reliance on rescue analgesics, further minimizing PONV risk. While the study by Kusderci et al. investigated the combination of SFIPB with IPACK, they found no additional benefit in opioid consumption or pain scores compared to SFIPB alone [14]. This is consistent with our observation that SFIPB, even as a standalone intervention, provides robust analgesia, particularly in reducing anterior knee pain during mobilization. Additionally, the role of SFIPB in combination with other regional anesthesia techniques, such as IPACK, warrants further investigation. While our study and Kusderci et al. found no additive benefit, tailored approaches based on individual patient profiles and surgical characteristics could provide new insights into optimizing multimodal analgesia strategies.

Although studies on the use of SFIPB in knee surgery are limited, its application in hip surgery has been widely investigated [15]. In these studies, the optimal balance between LA dose and volume to provide effective analgesia while preserving motor function remains unclear [16]. A combined radiological and cadaveric study demonstrated that a total volume of 40 mL was sufficient to encompass the femoral nerve, obturator nerve, and lateral femoral cutaneous nerve based on CT imaging and dissection data [17, 18]. Similarly, Kantakam et al., in their cadaveric dose-finding study, reported that the minimum effective volume in 90% of cases (MEV90) to block all three nerves of the lumbar plexus was approximately 62.5 mL [19]. A comprehensive radiological-cadaveric analysis by Ten Hoope et al. revealed that even with suprainguinal insertion and advanced cranial needle positioning using hydrodissection, obturator nerve involvement remained unreliable, being achieved in only 1 of 34 cases despite extensive cranial injectate spread [20]. This inconsistency likely stems from the anatomical trajectory of the obturator nerve, which traverses outside the fascia iliaca compartment, suggesting that SFIPB provides reliable coverage predominantly for the femoral and lateral femoral cutaneous nerves. These findings substantiate our approach in using a high-volume, low-concentration regimen, aimed at optimizing cranial diffusion while minimizing motor blockade.

This study has some limitations. First, the sample size, while adequate for detecting significant differences in opioid consumption, may limit the generalizability of secondary outcomes such as rehabilitation test results. Second, the use of a single-center design could introduce bias due to variations in surgical and anesthesia techniques. Future multicenter, randomized controlled trials with larger cohorts are warranted to confirm these findings and explore the long-term benefits of SFIPB in TKA. Finally, there remains a lack of consensus in the literature regarding the optimal local anesthetic dose, concentration, or the use of adjuncts for these techniques. Variations in volume, dose, and concentration can yield different outcomes and pain control efficacy. Furthermore, no block targeting posterior knee pain was applied in either group. The integration of this block with modalities specifically addressing posterior knee pain, such as the IPACK block, could potentially lead to more effective outcomes.

## Conclusion

In conclusion, this study underscores the advantages of SFIPB over PAI in TKA. By reducing opioid consumption and associated side effects while supporting early mobilization, SFIPB represents a promising addition to multimodal analgesia protocols. Its ability to deliver effective pain relief without compromising motor function. Future research should aim to refine its application and evaluate its integration into broader perioperative care pathways.

## Data Availability

No datasets were generated or analysed during the current study.
